# Para-hydroxyphenylpyruvate inhibits the pro-inflammatory stimulation of macrophage preventing LPS-mediated nitro-oxidative unbalance and immunometabolic shift

**DOI:** 10.1371/journal.pone.0188683

**Published:** 2017-11-27

**Authors:** Rosella Scrima, Marta Menga, Consiglia Pacelli, Francesca Agriesti, Olga Cela, Claudia Piccoli, Antonella Cotoia, Alessandra De Gregorio, Julia V. Gefter, Gilda Cinnella, Nazzareno Capitanio

**Affiliations:** 1 Department of Clinical and Experimental Medicine, University of Foggia, Foggia, Italy; 2 Laboratory of Pre-Clinical and Translational Research, IRCCS CROB, Rionero in Vulture, Potenza, Italy; 3 Department of Medical and Surgical Sciences, University of Foggia, Foggia, Italy; 4 Department of Critical Care Medicine, University of Pittsburgh School of Medicine, Pittsburgh, United States of America; University of Insubria, ITALY

## Abstract

Targeting metabolism is emerging as a promising therapeutic strategy for modulation of the immune response in human diseases. In the presented study we used the lipopolysaccharide (LPS)-mediated activation of RAW 264.7 macrophage-like cell line as a model to investigate changes in the metabolic phenotype and to test the effect of p-hydroxyphenylpyruvate (pHPP) on it. pHPP is an intermediate of the PHE/TYR catabolic pathway, selected as analogue of the ethyl pyruvate (EP), which proved to exhibit antioxidant and anti-inflammatory activities. The results obtained show that LPS-priming of RAW 264.7 cell line to the activated M1 state resulted in up-regulation of the inducible nitric oxide synthase (iNOS) expression and consequently of NO production and in release of the pro-inflammatory cytokine IL-6. All these effects were prevented dose dependently by mM concentrations of pHPP more efficiently than EP. Respirometric and metabolic flux analysis of LPS-treated RAW 264.7 cells unveiled a marked metabolic shift consisting in downregulation of the mitochondrial oxidative phosphorylation and upregulation of aerobic glycolysis respectively. The observed respiratory failure in LPS-treated cells was accompanied with inhibition of the respiratory chain complexes I and IV and enhanced production of reactive oxygen species. Inhibition of the respiratory activity was also observed following incubation of human neonatal fibroblasts (NHDF-neo) with sera from septic patients. pHPP prevented all the observed metabolic alteration caused by LPS on RAW 264.7 or by septic sera on NHDF-neo. Moreover, we provide evidence that pHPP is an efficient reductant of cytochrome *c*. On the basis of the presented results a working model, linking pathogen-associated molecular patterns (PAMPs)-mediated immune response to mitochondrial oxidative metabolism, is put forward along with suggestions for its therapeutic control.

## Introduction

Recent studies have highlighted the tight link between metabolic state and phenotype of immune-competent cells both in the innate and acquired immunity [1–4]. The emerging concept is that metabolism not only sustains diverse immune cell phenotypes as a consequence of alterations in cellular signalling, but also feeds back and alters signalling to drive the immune-cell phenotype. A common feature of pro-inflammatory immune cells, such as effector lymphocytes, Tr1 regulatory T cells, M1 macrophages, mature dendritic cells, neutrophils and other granulocytes, is that they adopt a distinct metabolic signature termed “aerobic glycolysis” to support cellular biosynthetic processes: that is, glucose metabolized to lactate in the presence of abundant oxygen. Aerobic glycolysis is strategic to cells engaged in robust growth and proliferation because it provides fast ATP production (albeit with low efficiency) and biosynthetic precursors that are essential for the synthesis of nucleotides, amino acids, and lipids. Such a metabolic phenotype closely resembles what, since long observed in many cancer cells, known as "Warburg effect". Conversely, other immune cell subsets, such as naïve lymphocytes, memory T cells, FoxP3+ regulatory T cells, M2 macrophages, rely on oxidative metabolism [5].

In this context mitochondria are being increasingly recognized as signalling organelles for both the maintenance and establishment of immune cell phenotypes [6]. In addition to the recognized role in cellular bioenergetics mitochondria function as hubs integrating multiple innate immune signalling [7]. Mitochondria are also a major source and target of reactive oxygen and nitrogen species, establishing oxidative unbalance in various context, including the response to bacterial infection. On this basis, targeting the mitochondrial oxidative metabolism is becoming an attractive therapeutic strategy for modulation of the immune response in human diseases.

Ethyl pyruvate (EP), the ester derivative of pyruvate, exhibits a variety of pharmacological effects including: down-regulation of the secretion of pro-inflammatory cytokines; enhanced anti-tumor immunity; amelioration of redox-mediated damage to cells and tissues; inhibition of apoptosis (under some circumstances) or promotion of apoptosis (under other circumstances); and support of cellular ATP synthesis [8–10]. Some of the pharmacological effects of EP are likely to be linked to its function as ROS and RNS scavenger, a property elicited by its α-cheto carboxylate moiety [11,12]. However, in spite of the protecting effect of EP in several animal models of critical illness including myocardial or mesenteric ischemia/reperfusion injury, sepsis, and hemorrhagic shock, its benefits in critical care patients has not been confirmed [13]. A search of alternative EP-related chemical compounds led us to characterize the effect of p-hydroxyphenylpyruvate (pHPP), an intermediate of the PHE/TYR catabolism, on a rat model of profound hemorrhagic shock [14]. The results obtained showed that a small volume administration of a pHPP solution significantly prolonged survival of rats subjected to 50% of blood withdrawal. Moreover, in vitro analysis on cultured endothelial cell lines unveiled that pHPP prevented cell death under stressing conditions. This effect was ascribable to antioxidant properties toward mitochondria-generated ROS and to improvement of the mitochondrial respiration and related oxidative phosphorylation (OxPhos) [14].

In the presented *in vitro* study we have extended our analysis on the pharmacological properties of pHPP in the context of a well-established pro-inflammatory model constituted by lipopolysaccharide (LPS)-stimulated macrophage cell line. The results obtained clearly indicate for pHPP a robust anti-inflammatory activity counteracting the metabolic rewiring of LPS-stimulated macrophage and the related ensued pro-inflammatory pathways.

## Materials and methods

### 2.1 Cell cultures

RAW 264.7 macrophage-like cell line (obtained from ATCC, Manassas, VA) and neonatal normal human dermal fibroblasts (NHDF-neo) (obtained from Lonza Group Ltd., Basel, Switzerland) were maintained in a humified 5% CO_2_ incubator at 37°C in Dulbecco’s modified Eagle medium (DMEM—low glucose (i.e. 5.5 mM)) supplemented with 10% FBS (Hyclone Laboratories, Logan, UT), 1% penicillin/streptomycin, 1% pyruvate. Cells were passed once a week and used for experiments at 80–90% confluence. All cell culture lines were routinely tested on a monthly basis for *Mycoplasma* contamination using the Lonza MycoAlert^®^ Mycoplasma Detection Kit (Rockland, ME). RAW 264.7 cell line and NHDF-neo were never used at passages higher than 15 and 8 respectively.

### 2.2 Materials

para-Hydroxylphenylpyruvate (pHPP), ethylpyruvate (EP), dichloroacetate (DCA), rotenone, oligomycin, carbonyl cyanide 4-(trifluoromethoxy)phenylhydrazone (FCCP), carbonyl cyanide m-chlorophenylhydrazone (CCCP) were purchased from Sigma-Aldrich Chemical Co. All the other chemicals were of the highest purity grade commercially available.

### 2.3 Cell viability assay

For MTS assay, cells were seeded in a 96-well culture plate in DMEM supplemented without or with graded concentrations of pHPP for 24h. Cell viability was measured using solutions of the novel tetrazolium compound (3-(4,5-dimethylthiazol-2-yl)-5-(3-carboxymethoxyphenyl)-2-(4-sulfophenyl)-2H-tetrazolium, inner salt (CellTiter 96^®^ AQueous MTS Reagent Powder, Promega) and the electron coupling reagent, phenazine methosulfate, PMS (Sigma Aldrich, Saint Louis, MO, USA). MTS is bioreduced by cells into a formazan product that is soluble in tissue culture medium. The absorbance of the formazan at 490 nm was measured directly from 96-well assay plates using the Plate Reader (das srl, Italy) with a reference filter at 630 nm. All measurements were performed in triplicate for each assay.

### 2.4 Nitrite/nitrate and IL-6 measurements

RAW 264.7 macrophage-like cells were plated in 6-well plates and used the following day. Cells were stimulated by adding 10 ng/ml *Escherichia coli* LPS (serotype O111:B4) in the presence or absence of graded concentrations of the tested compounds (EP, pHPP). Nitrite/nitrate and IL-6 concentrations were measured after 18 h in cell supernatants using a commercially available Griess reaction kit (Oxis International, Portland, OR) and the commercially available Quantikine Immunoassay Kit for IL-6 (R&D Systems, Minneapolis, MN) respectively.

### 2.5 q-RT-PCR analysis of iNOS

RAW 264.7 cells were harvested at 24 h in 1 ml of TRI-Reagent as directed by the manufacturer (Molecular Research Center, Inc.). Bromochloropropane was used for the extraction. The final RNA pellet was dissolved in nuclease-free water and quantified using a GeneQuant pro UV spectrophotometer (GE Healthcare). Extracted RNA (1 μg /reaction) was converted to single-stranded cDNA in a 20 μl reaction using the Reverse Transcriptase System Kit (Promega) as directed by the manufacturer. The mixture was heated to 70°C for 10 min, maintained at 42°C for 30 min, and then heated to 95°C for 5 min using a Gene Amp PCR System 9700 (Applied Biosystems, Foster City, CA). TaqMan Gene Expression Assays for mouse iNOS and 18S RNA (endogenous control) and real-time PCR reagents were from Applied Biosystems (Foster City, CA). The oligonucleotide primers used were: (F)5′-CCCTTCCGAAGTTTCTGGCAGCAGC-3′ and (R)5′-GGCTGTCAGAGAGCCTCGTGGCTTTGG-3′ for the mouse macrophages iNOS; (F)(5′-ATGCCATCCTGCGTCTGGACCTGGC-3′ and (R)5′-AGCATTTGCGGTGCACGATGGAGGG-3′ for the mouse β-actin. Reaction mixtures for PCR were assembled as follows: 10 μl TaqMan Universal PCR Master Mix, 1 μl of each Gene Expression Assay mix, 1 μl cDNA template and 7 μl of water. The real time RT-PCR analysis was performed using ABI Prism 7900HT (University of Pittsburgh Genomics and Proteomics Core Laboratories) by incubating at 50°C for 2 min, 95°C for 10 min, 95°C for 15 s, and 60°C for 1 min; the two final steps were repeated for 40 cycles. Each sample was assayed in duplicate and the values were averaged. A ΔΔCt relative quantification method was used to calculate mRNA levels in the samples. Results were first normalized relative to 18S mRNA expression levels and than to the control (calibrator).

### 2.6. Imaging of intracellular NO and peroxide production

RAW 264.7 cells, plated in 6-well plates, were treated with 10 ng/ml LPS with or without 3 mM pHPP for 18 h. Thereafter, either 10 μM DAF-FM (4-amino-5-methylamino-2',7'-difluorofluorescein diacetate) or 5 μM DCF-DA (2',7'-dichlorofluorescein diacetate) were added to detect RNS (mainly NO) or ROS (mainly peroxides) respectively. Specificity of the NO-probe was verified by treating the LPS-challenged cells with 1 mM of the NOS pan-inhibitor L-NAME (L-N^G^-nitroarginine methyl ester). Imaging of the intracellular fluorescent probes was performed by the EVOS Cell Imaging System (Thermo Fisher). Quantitative analysis of the digitalized images was carried out with ImageJ *(*https://imagej.nih.gov/ij/).

### 2.7. Respirometry

Oxygen consumption rate in intact cells suspension was measured by high resolution oxymetry (Oxygraph-2K, Oroboros) in thermostatically controlled tween-chambers (T = 37°C) equipped with a stirring device and a gas-tight plug equipped with a narrow port enabling addition by microsyringe. Cultured RAW 264.7 and NHDF-neo were detached from the plate by trypsinization, washed in PBS and suspended at 2–4 x10^6^ cells/ml in 50 mM KH_2_PO_4_, 10 mM Hepes, 1mM EDTA, pH7.4 in both the oxymeter chambers. Respiration of untreated and immunostimulated cells samples (± co-incubation with pHPP) were assayed in parallel. After attainment of the stationary resting oxygen consumption rate, 1 μg oligomycin/ml was added followed 5 min later by addition of 0.5 μM FCCP and finally of 2 μM rotenone + 1 μM antimycin A.

### 2.8. Metabolic flux analysis

Oxygen consumption rate (OCR) and extra-cellular acidification rate (ECAR) were measured in adherent RAW 264.7 cells with a XF96 Extracellular Flux Analyzer (Seahorse Bioscience, Billerica, MA, USA). For OCR analysis, after replacing the growth medium with 180 μl of bicarbonate-free DMEM supplemented with 10 mM glucose 2 mM L-glutamine and 1 mM sodium pyruvate pre-warmed at 37°C, cells were preincubated for 45 min before starting the assay procedure. After measuring basal respiration, Oligomycin (1 μM), carbonyl cyanide m-chlorophenylhydrazone (0.8 μM), and rotenone + antimycin A (1 μM + 1 μM) were injected into each well sequentially to assess respectively coupling of respiratory chain, maximal and non-mitochondrial oxygen consumption. For ECAR analysis, after replacing the growth medium with 180 μl of bicarbonate-free DMEM pre-warmed at 37°C, cells were preincubated for 45 min before starting the assay procedure. Glycolytic flux (basal glycolysis, glycolytic capacity, and glycolytic reserve) was analyzed by the sequential addition of 10 mM glucose, 1 μM oligomycin, and 100 mM 2-deoxyglucose. OCR and ECAR values were normalized to protein content in each well, determined with BCA assay.

### 2.9. Mitochondrial respiratory complexes activity

The specific activities of the complexes I and IV of the mitochondrial respiratory chain were assayed spectrophotometrically on frozen–thawn and ultrasound-treated cells as previously described [15,16].

### 2.10. Spectrophotometric analysis of cytochrome c

Time-course of absorbance at 550 nm (ε = 21 mM^-1^cm^-1^) and UV-Vis spectra of horse heart cytochrome c (Sigma-Aldrich Chemical Co) were carried out by a split-beam spectrophotometer (Perkin-Elmer λ40); ε_red,550nm_ = 29.5 mM^-1^cm^-1^, ε_red-ox,550nm_ = 21.1 mM^-1^cm^-1^.

### 2.11. Isolation of mitochondria

Mitochondria were isolated from beef heart homogenate by differential centrifugation in 0.25 M sucrose, 5 mM Hepes, 1 mM EDTA, 5 mM MgCl_2_ as described in [17] and stored frozen at -80°C.

### 2.12. Western blotting analysis

Aliquots containing 40 μg of proteins from each cell lysate were subjected to SDS polyacrylamide gel electrophoresis and transferred to a polyvinylidene difluoride membrane (Bio-Rad Laboratories; Hercules, CA, USA) using Trans Blot Turbo Transfer System. Membranes were probed with the following primary antibodies: HIF-1α (1:1000; Santa Cruz Biotechnology Inc., sc-13515) and β-actin (1:5000; SIGMA Aldrich, St. Louis, MO, USA). After incubation with corresponding suited horseradish peroxidase-conjugated secondary antibody (1:2500; Cell Signaling Technology) signals were developed using the enhanced chemiluminescence kit (ClarityTM Western ECL Substrate, Bio-Rad), acquired by ChemiDoc Imaging System XRS + (BioRad) and analyzed for densitometry with the ImageJ Lab 4.1 software.

### 2.13. Septic patients’ serum collection

Eight adult (> 18 years) septic shock patients, as defined by The Third International Consensus Definition for Sepsis and Septic Shock (Sepsis-3) [18] were enrolled. All patients had to exhibit positive blood cultures or a defined focus of infection (e.g. peritonitis or pneumonia) requiring vasopressor therapy (> 0.5 μ/kg/min norepinephrine) to maintain mean arterial pressure greater than 65 mm Hg despite adequate volume expansion. All patients were required to have procalcitonin levels > 2 ng/ml, CRP > 10 mg/ml and ScVO_2_ ≥ 70% [19,20]. For serum separation, venous blood samples (10 ml) were withdrawn 24 h after the diagnosis of sepsis from each informed patient in Vacutainer^®^, centrifuged at 500 g for 10 min at 25°C and then the supernatants were harvested and directly used following decomplementation (56°C for 30 min). Blood from eight healthy volunteers (control group) was subjected to the same treatment. NHDF-neo cells were incubated in DMEM supplemented with 1:10 (v/v) of either normal or septic sera for 18 h in the absence or in the presence of 3 mM pHPP. For the utilization of patients’ samples specific approval of the Local Ethical Committee was obtained for this study (Opedali Riuniti University Hospital cod. XX/CE/2014). Written informed consent was obtained from all participants.

### 2.14. Statistical analysis

Data are presented as means ± S.E.M. and analyzed using the variance analysis test (ANOVA) followed by a post hoc Bonferroni test or the two-tailed student t-test. A value of P<0.05 was set as statistically significant.

## Results

**Fig 1:**
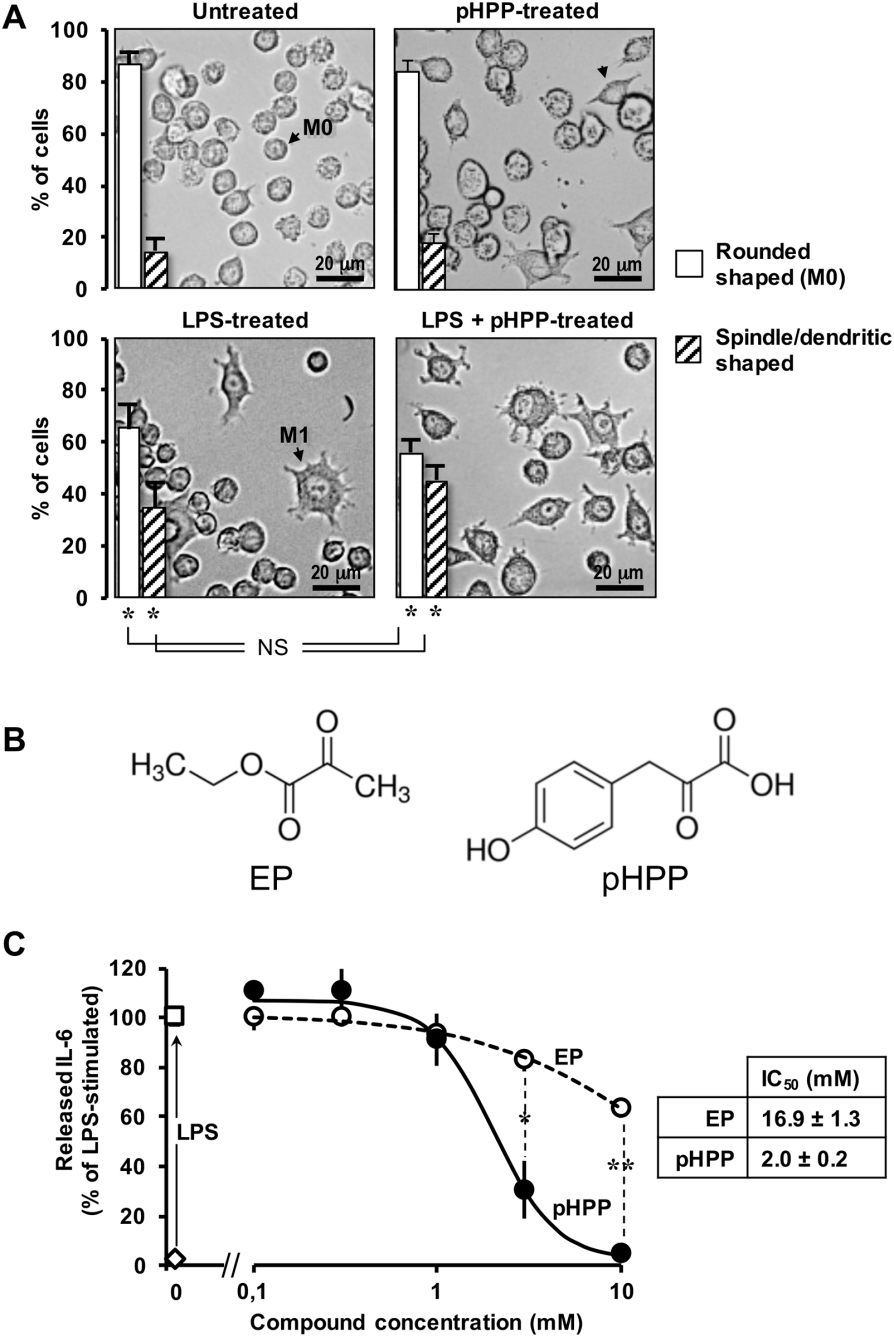
Effect of graded concentrations of EP and pHPP on IL-6 release in LPS-treated RAW 264.7 macrophage cell line. **(A)** Cultured cells were treated with 10 ng/ml LPS for 18 h and morphological changes analyzed by light transmission microscopy. Images show representative micrographs of untreated and LPS-stimulated cells (± 3 mM pHPP). The histograms in the pictures show the morphometric analysis carried out on images collected from 3–4 independent experiments averaging (± SEM) at least 5 different optical fields for each condition in each biological replicate; *: P<0.05 vs untreated ± 3 mM pHPP cells. See text for further details. **(B)** Cultured cells were treated with LPS as in (A) in the absence and in the presence of the indicated concentrations of either EP or pHPP and assayed for the IL-6 released in the supernatant as described in Materials and Methods. The values are means ± SEM of the percentages of untreated sample and refer to 3 independent experiments under each condition; *: P<0.005, **: P <0.0001.

The murine macrophage RAW 264.7 cell line is a well-established *in vitro* model used to test the immunomodulatory activity of compounds following immunostimulation by LPS [21]. Fig 1A shows that treatment of the RAW 264.7 cell line with 10 ng LPS/ml for 18 h induced a change in the cell phenotype highlighted by morphological changes leading to progressive polarization of macrophages from the undifferentiated rounded-shaped M0 state to the activated dendritic-shaped M1 state. Consistently, LPS-stimulation of the RAW 264.7 cell line resulted in release of the pro-inflammatory cytokine IL-6 (Fig 1C). pHPP or ethyl pyruvate (EP) (Fig 1B) prevented dose-dependently IL-6 production in LPS-stimulated macrophages albeit with different efficiency. Indeed, while 10 mM pHPP fully suppressed IL-6 production, a similar concentration of EP caused only about 40% decrease of the cytokine release (Fig 1C). The IC_50_ estimated from non-linear fit of the experimental points was 16.9 and 2.0 mM for EP and pHPP respectively. The limited solubility of EP impeded to test higher concentration of this compound. In spite of its marked inhibition on the release of the pro-inflammatory cytokine, pHPP-treatment had no statistical significant impact on the LPS-induced morphological changes of RAW 264.7 cell line (Fig 1A). When cells were incubated with pHPP alone no significant morphological changes were observed except for the appearance of a few spindle-like/elongated cells (Fig 1A).

**Fig 2:**
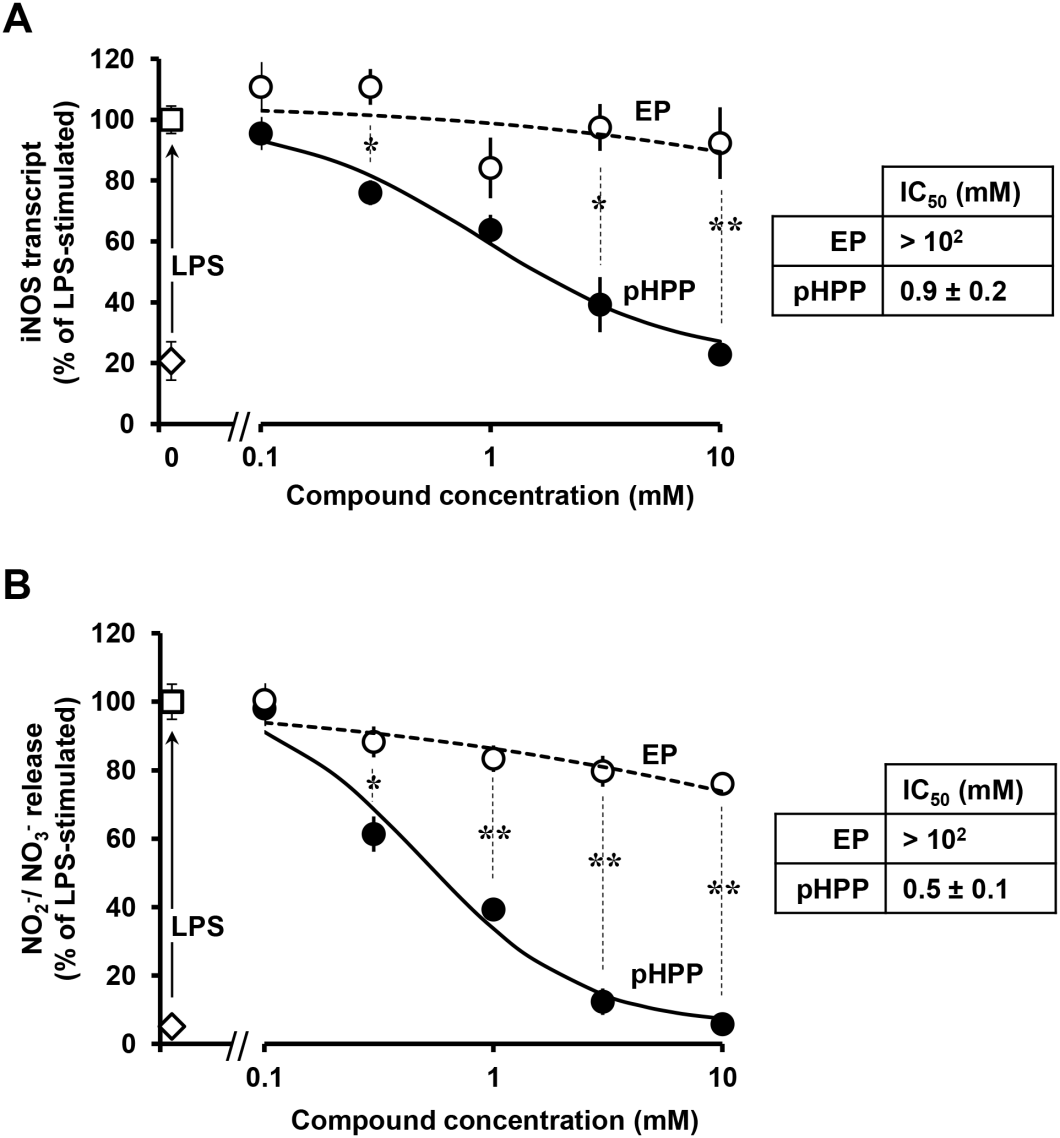
Effect of graded concentrations of EP and pHPP on iNOS expression and nitrite/nitrate release in LPS-treated RAW 264.7 macrophage cell line. Cultured cells were treated with 10 ng/ml LPS for 18 h in the absence and in the presence of the indicated concentrations of either EP or pHPP and assayed for iNOS mRNA level in cell lisates **(A)** and for the nitric oxide (NO) derivatives nitrite/nitrate in the supernatant **(B)**. The values in (A) and (B) are means ± SEM of 3 independent experiments under each condition; *: P<0.05, **: P <0.01, ***: P <0.001.

A well-established feature of LPS-stimulated macrophage is the transcriptional activation of the inducible nitric oxide synthase (iNOS) [22]. Therefore, next we tested the effect of pHPP on the LPS-mediated induction of iNOS expression and NO production. Fig 2A shows that LPS-stimulation of RAW 264.7 macrophages elicited a large increase in transcription of the *NOS2* gene (coding for iNOS) and in consequential production of NO assessed as NO_2_^-^/NO_3_^-^ formation (Fig 2B). Grading amounts of pHPP prevented progressively both iNOS expression and NO production with an estimated IC_50_ of 0.9 and 0.5 mM respecively. Conversely, though the limited solubility of EP hindered a precise estimation of its IC50, nevertheless a much lower inhibitory effect was evident on both iNOS expression and NO production in LPS-treated cells, as compared with pHPP.

**Fig 3:**
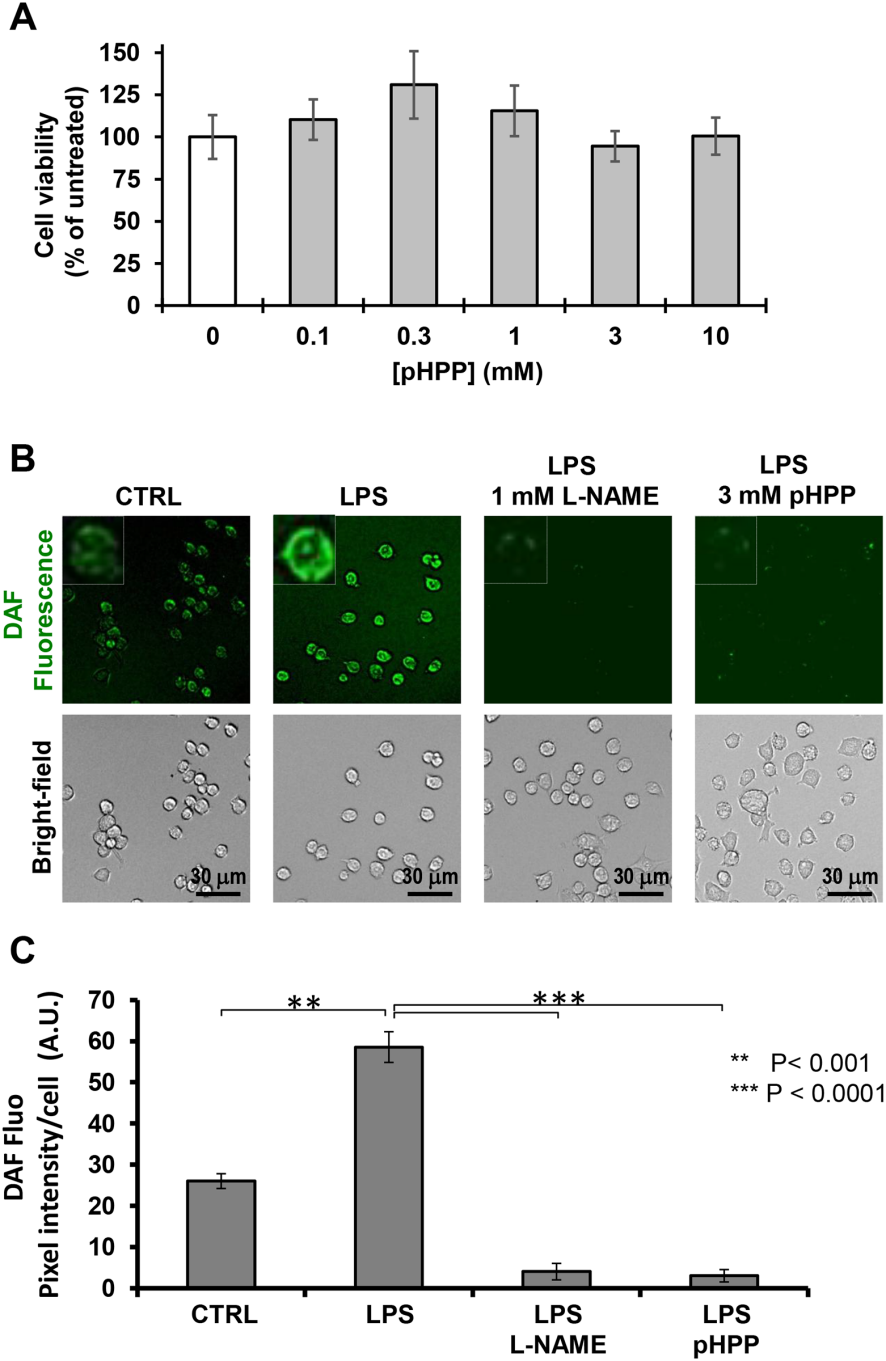
Fluorescence microscopy imaging of cellular production of NO in LPS-treated RAW 264.7 macrophage cell line and effect of pHPP. Cultured cells were treated with LPS as in Fig 1 in the absence or presence of either 1 mM L-NAME or 3 mM pHPP. Then after cells were supplemented with DAF-DA and visualized by fluorescence microscopy as detailed under Materials and Methods. (**A**) Representative fluorescence images along with the corresponding bright-fields. (**B**) Intracellular fluorescence quantification performed on digitalized images by ImageJ. The bars are means ± SEM of 4 independent biological replicates obtained by averaging the intracellular pixel intensity obtained from at least 5 different fields for each condition. The statistical significance of the difference is also shown.

From this point ahead we decided to characterize more in depth the specific properties of pHPP. The testing concentration of 3 mM of the compound was chosen to minimize solubility-related harms. The MTS-based citotoxicity assay showed that 3 mM pHPP caused no change in cell viability (Fig 3A).

The marked effect of pHPP on the NO production was was further confirmed by microscopy imaging by the intracellular fluorescent probe DAF [23]. Fig 3B and 3C show that the overproduction of NO in LPS-stimulated RAW 264.7 cells was almost completely abrogated by 3 mM pHPP; similar result was obtained by the NOS pan-inhibitor L-NAME thus confirming the specificity of the probe.

**Fig 4:**
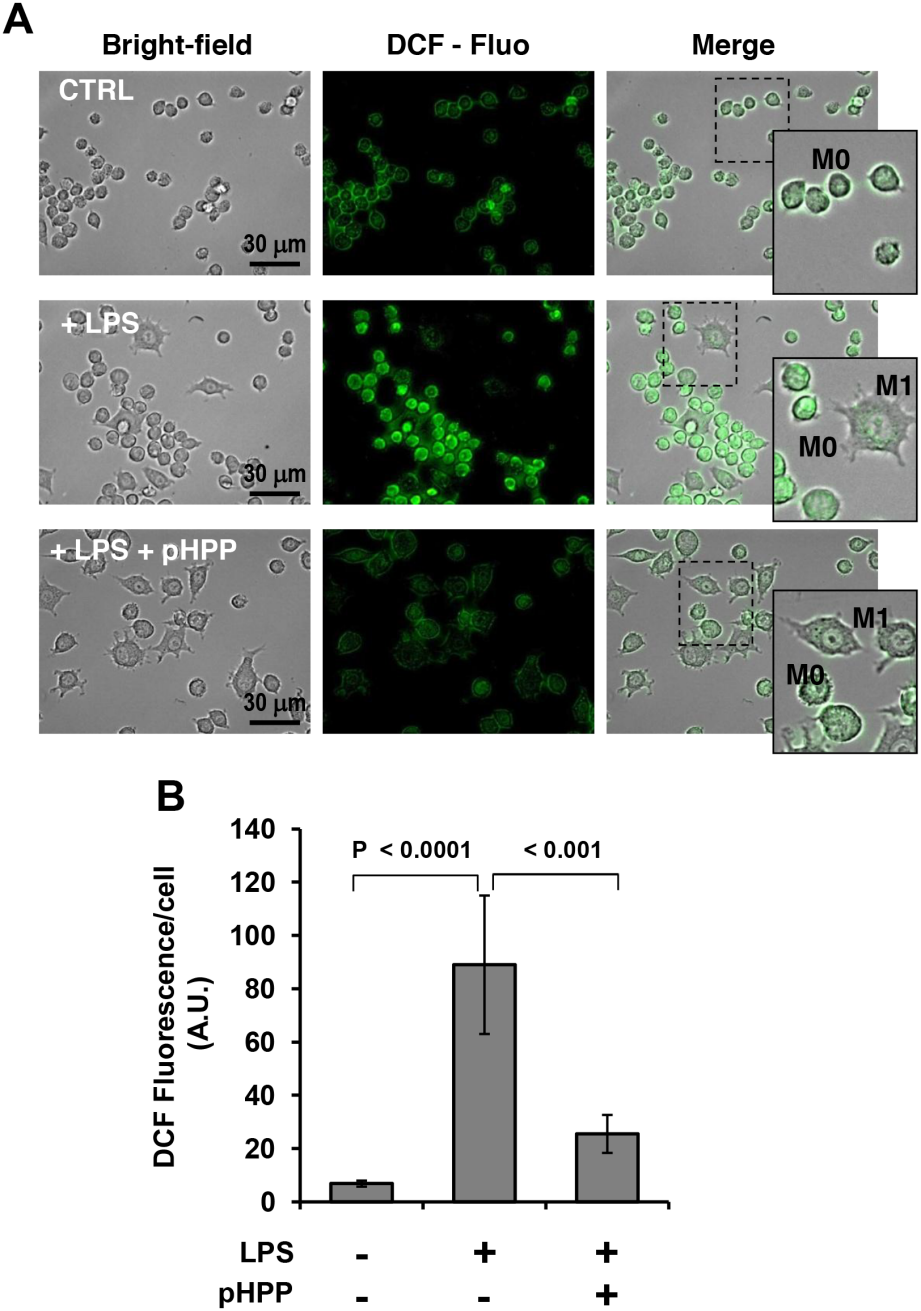
Fluorescence microscopy imaging of cellular production of peroxides in LPS-treated RAW 264.7 macrophage cell line and effect of pHPP. Cultured cells were treated with LPS as in Fig 1 in the absence or presence of 3 mM pHPP. Then after cells were supplemented with DCF-DA and visualized by fluorescence microscopy as detailed under Materials and Methods. (**A**) Representative fluorescence images along with the corresponding bright-fields and image merge; digital magnifications of selected areas is also shown. (**B**) Intracellular fluorescence quantification performed on digitalized images by ImageJ. The bars are means ± SEM of 4 independent biological replicates obtained by averaging the intracellular pixel intensity obtained from at least 5 different fields for each condition. The statistical significance of the difference is also shown.

Along with the generation of NO, LPS stimulation of macrophages caused also reactive oxygen species (ROS) overproduction [24]. Accordingly, Fig 4 shows a large change in the intracellular redox tone of LPS-treated RAW 264.7 using the redox-sensitive fluorescent probe DCF. Though DCF was found to react with a variety of reactive redox-active species nevertheless its commonly considered a sensor of peroxides [25]. As for NO, 3 mM pHPP largely prevented production of *bona fide* ROS in LPS-stimulated RAW 264.7 macrophages.

**Fig 5:**
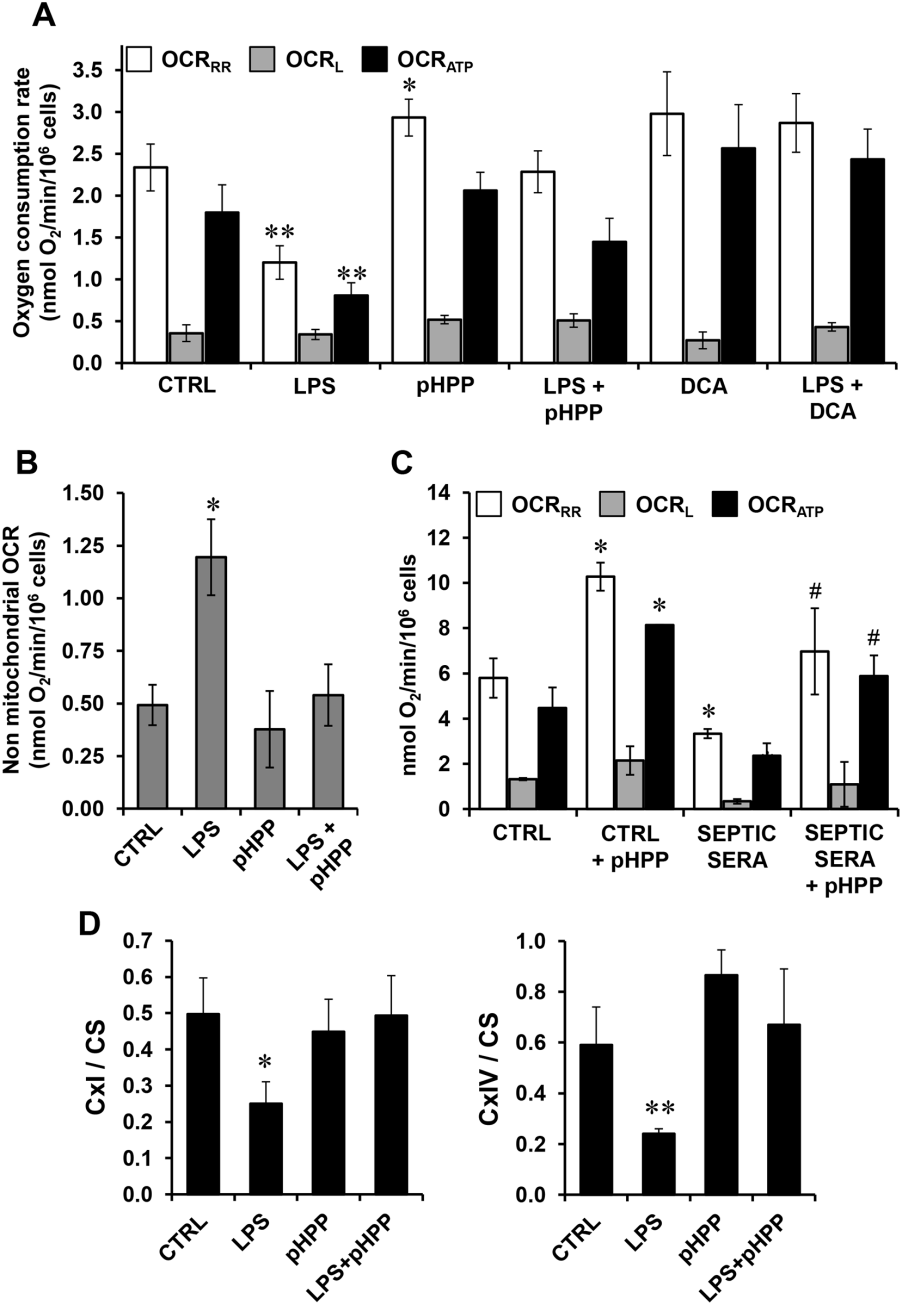
Measurement of mitochondrial respiratory chain activity in LPS-treated RAW 264.7 murine macrophage cell line and in septic sera-treated NHDF human fibroblasts and effect of pHPP. **(A)** Respirometric analysis of intact RAW 264.7 cells. Cultured cells were incubated for 18 h with the indicated compounds at the following concentrations: 10 ng/ml LPS, 3 mM pHPP, 2.5 mM DCA. The bars indicate the oxygen consumption rate (OCR) recorded by high-resolution polarimetry, corrected for 2 μM rotenone + 1 μM antimycin A-insensitive activity and normalized to the cell number; means ± SEM of 4–6 independent biological replicates under each condition. OCR_RR_, activity measured under resting respiration; OCR_L_, activity measured in the presence of oligomycin; OCR_ATP_, difference between OCR_RR_ and OCR_L_ (see Materials and methods and Results for their functional meaning). *, P < 0.05 vs the corresponding OCRs of control (CTRL); **, P < 0.01 vs the corresponding OCRs of all the other conditions. (**B**) Mitochondria-unrelated OCR. The histogram shows the OCR recorded after addition of rotenone + antimycin A; means ± SEM of 6 independent biological replicates under each condition; *, P < 0.05 vs CTRL. **(C)** Respirometric analysis of intact human NHDF. Cultured cells were incubated for 18 h with media supplemented with 1/10 volume of sera isolated from either healthy or septic patients without or with 3 mM pHPP as indicated. The bars indicate the oxygen consumption rate (OCR) recorded by polarimetry and normalized to the cell number and are means ± SEM of 4 independent biological replicates under each condition. *, P < 0.05 vs CTRL; ^#^, P < 0.05 vs septic sera-treated cells. **(D)** Activity of mitochondrial respiratory chain complexes. RAW 264.7 cell were incubated with the indicated compounds as in panel (A). Then after cells were lysated and the specific enzymatic activity of the NADH dehydrogenase (CxI) and cytochrome c oxidase (CxIV) assessed by spectrophotometric assays under condition of saturating substrates as detailed under Materials and Methods. The measured activities were normalized to the citrate synthase activity. The bars indicate the means ± SEM of 4 independent biological replicates under each condition. *, P < 0.05 vs all the other conditions; **. P < 0.01 vs all the other conditions.

Since NO and ROS are potential inhibitors of the mitochondrial respiratory chain complexes [26], we measured the oxygen consumption rate in intact RAW 264.7 cells by high resolution respirometry. Fig 5A illustrates the results of a systematic analysis showing that LPS-stimulation of RAW 264.7 resulted in a significant 50% reduction of the oxygen consumption rates measured under condition of resting respiration (OCR_RR_) as compared with untreated or pHPP-treated cells. In the presence of the F_0_F_1_ ATP-synthase inhibitor oligomycin the OCR was largely depressed in all the conditions. This respiratory activity is an indirect measurement of the passive proton leak ensued by the built-up of the mitochondrial membrane potential no longer collapsed by the ATP-synthase activity (OCR_L_). The difference between the OCR_RR_ and the OCR_L_ is taken as an indirect measure of the effective mitochondrial oxygen consumption utilized to make ATP synthesis (OCR_ATP_). The OCR_ATP_ in LPS-challenged cells was significantly depressed thereby indicating a severe OxPhos failure in immune-stimulated RAW 264.7 cells. Notably, when LPS-stimulated cells were treated with pHPP both the decrease of OCR_RR_ and OCR_ATP_ was largely prevented displaying activities comparable with untreated cells. A small but statistically significant increase of the OCR was observed treating unstimulated cells with pHPP. Results similar to those attained by pHPP were observed co-incubating LPS-stimulated RAW 264.7 with dichloroacetate (DCA), a known activator of the mitochondrial pyruvate dehydrogenase and effector of cell metabolism [27,28]. All the above reported OCRs were corrected for the rotenone+antimycin A-insensitive respiration and therefore attributable to the activity of the respiratory chain. Notably, the mitochondria-unrelated cellular OCR (i.e. the residual oxygen consumption in the presence of the respiratory chain inhibitors) resulted significantly higher in LPS-treated cells and was re-normalized to the control levels by pHPP (Fig 5B).

Next, we sought to verify if the pro-inflammatory background observed in septic patients could elicit inhibition of the mitochondrial respiratory activity as observed in LPS-stimulated RAW 264.7 and if this was sensitive to pHPP treatment. To these aim sera from a cohort of septic patients were supplemented to the culture medium of TLR4-expressing neonatal normal dermal human fibroblasts (NHDF-neo) [29] and their respiratory activity assessed after 18 h incubation; sera from healthy donors served as control. Fig 5C shows that treatment of NHDF-neo with septic serum caused a significant 50% reduction of the mitochondrial respiratory efficiency as compared with NHDF-neo treated with normal human sera. Importantly, co-incubation of septic sera with pHPP resulted in a respiratory profile comparable with that of control NHDF-neo if not slightly increased. To note, pHPP had a significant stimulatory effect of respiration also on NHDF-neo incubated with normal non-septic sera.

Complexes I and IV (NADH-dehydrogenase and cytochrome c oxidase respectively) of the respiratory chain are the major enzymatic steps controlling the overall respiratory flux [30]. Consistently measurements in LPS-stimulated RAW 264.7 of the enzymatic activity of both complexes I and IV (normalized to the citrate synthase activity) unveiled a significant inhibition of both, which was fully prevented by co-treatment with pHPP (Fig 5D).

**Fig 6:**
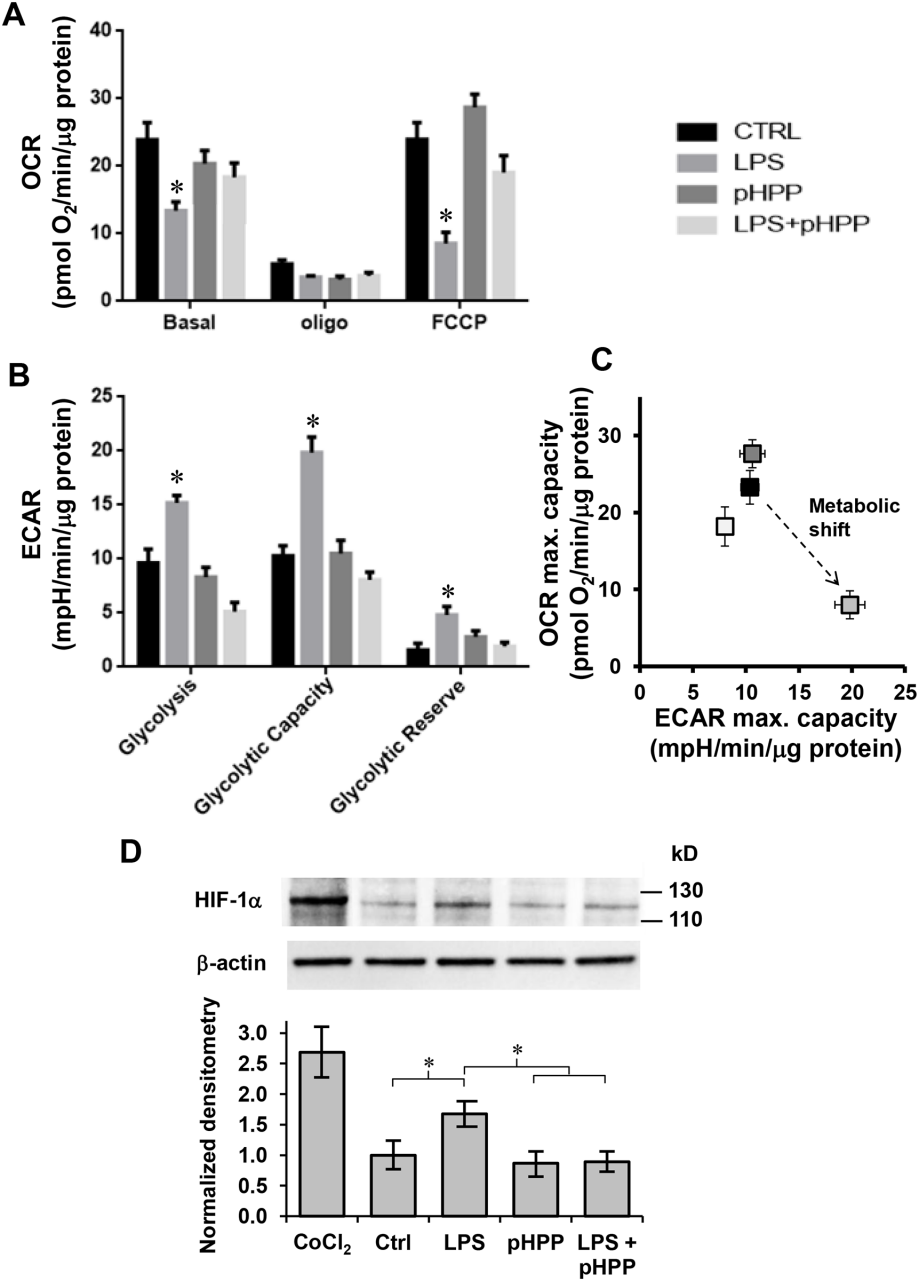
Metabolic flux analysis and expression of HIF-1α in LPS-treated RAW 264.7 macrophage cell line and effect of pHPP. Cells were seeded in a 96-wells cell culture microplate and incubated 18 h without or with 10 ng/ml LPS ± 3 mM pHPP (see the color code legend on the right). The histograms in **(A)** and **(B)** show the oxygen consumption rates (OCR) and the extra cellular acidification rates (ECAR) respectively assayed by the SeaHorse platform as described under Materials and Methods and normalized to the protein content of the cells removed from each well at the end of the assay. OCR in **(A)**: Basal, resting OCR; Oligo, OCR measured after the addition of the ATP synthase inhibitor oligomycin also referred as proton leak; FCCP, OCR measured after the addition of the uncoupler FCCP eliciting the maximal respiratory capacity. The OCR were corrected for the residual OCR measured after the addition of the CxI inhibitor rotenone (not shown). ECAR in **(B)**: Glycolysis, resting ECAR; Glycolytic Capacity, ECAR measure after the addition of oligomycin and FCCP and refers to the maximal glycolytic activity with the OxPhos inhibited; Glycolytic Reserve, difference between ECAR measured in the presence of oligomycin + FCCP and under resting conditions. The bars in (A) and (B) are means ± SEM of 3 independent experiment carried out in 3 technical replicates under each condition; *, P<0.05 vs all the other conditions. (**C**) Energy map obtained plotting the maximal ECAR and OCR capacity measured in (A) and (B) (same color code). **(D)** Immunoblotting of HIF-1α. Fourty micrograms of proteins from total protein extracts from untreated and compound-treated cells were subjected to SDS-PAGE and immunoblotted for detection of HIF-1α and β-actin, as loading control, as detailed in Materials and Methods. The experimental conditions for compounds treatments are as in panel (A). CoCl_2_ refers to a positive control carried out treating RAW 264.7 cells with 100 μM of the hypoxia-mimetic CoCl_2_ for 18 h. The upper panel shows a representative immunoblot, the lower histogram shows the densitometric values of HIF-1α normalized to β-actin as means ± SEM of 3 independent experiment; *, P < 0.05.

To confirm the above reported observations on the respiratory activity of RAW 264.7 cells we measured directly the metabolic fluxes in cultured cells by the SeaHorse technology [31]. As shown in Fig 6A the inhibitory effect of LPS on the rotenone-sensitive OCRs under basal and uncoupled states, and the preserving effect of pHPP were substantially confirmed. In addition, we assessed the extracellular acidification rates (ECAR) which is an indirect measurement of the glycolytic flux. Fig 6B shows that LPS-treated cells exhibited a significant increase of glycolysis which was fully prevented by pHPP. All together the presented observations clearly show that LPS-treatment caused rewiring of the main catabolic pathways depressing mitochondrial OxPhos meanwhile fostering glycolysis and that pHPP was able to largely prevent such a metabolic shift (Fig 6C).

The hypoxia-inducible factor 1 is a transcription factor controlling the expression of genes involved in the glycolysis under hypoxic conditions [32]. However, its normoxic activation has been demonstrated in macrophages and dendritic cells by pro-inflammatory stimuli [33,34]. Fig 6D shows that treatment of RAW 264.7 cells with either the hypoxia-mimetic CoCl_2_ or LPS induced normoxic stabilization of the transcription factor subunit alpha (HIF-1α) over the ground level in untreated cells. Co-treatment of LPS with pHPP resulted in no change in the basal level of the HIF-1α subunit.

**Fig 7:**
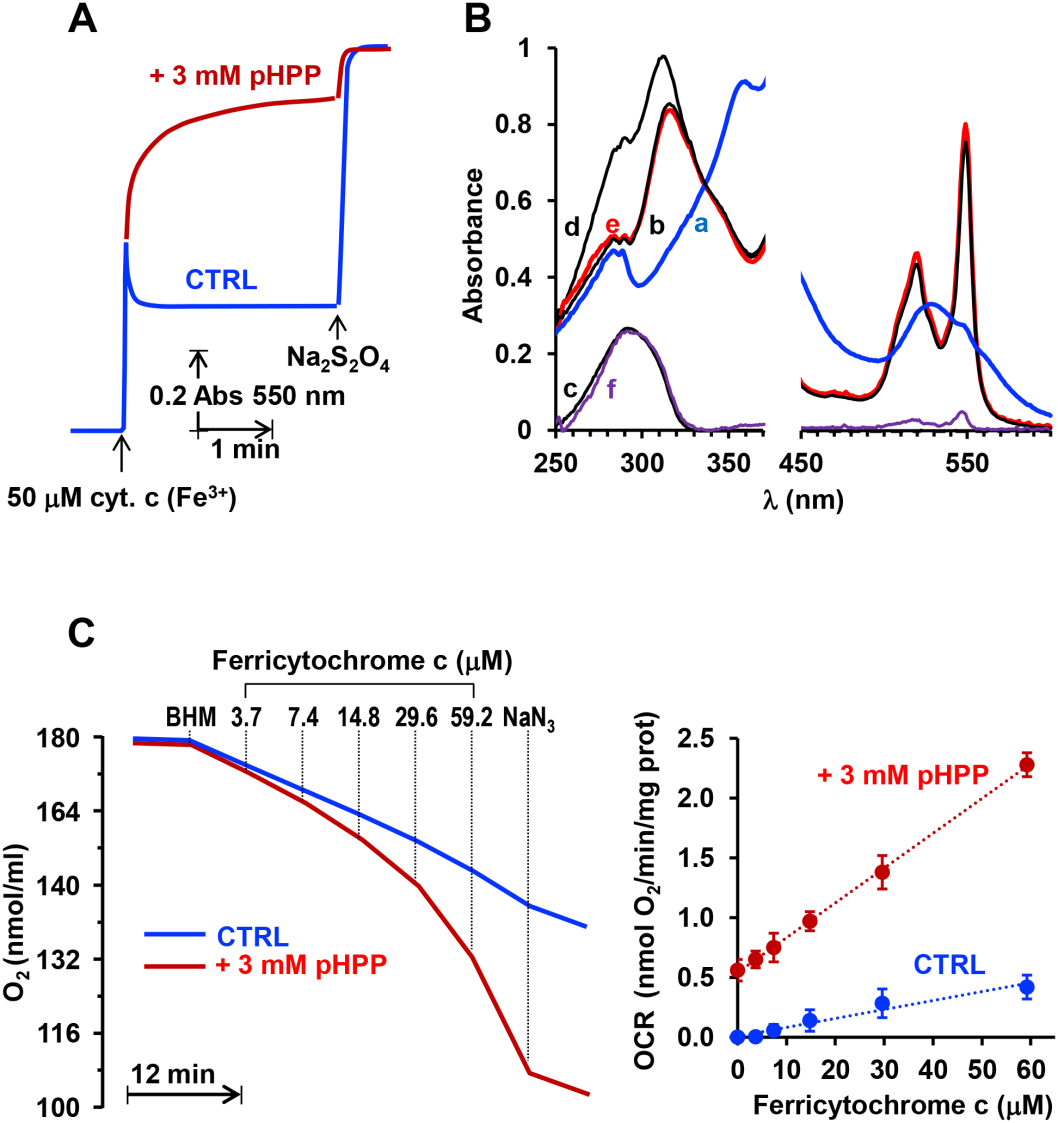
Redox properties of pHPP. (**A**) Spectrophotometric absorbance changes at 550 nm. Where indicated ferric cytochrome c was added to 0.25 M sucrose, 5 mM Hepes, 1 mM EDTA, 3 mM MgCl_2_, pH = 7.4 ± 3 mM pHPP; complete reduction of cytochrome c was attained by addition of a few grains of sodium dithionite (Na_2_S_2_O_4_). The time-course shown is representative of 3 independent experiments with and average t1/2 for cytochrome c reduction of 0.8 ± 0.05 sec. (**B**) Spectra analysis in the UV and visible range. (a), 50 μM ferric cytochrome c; (b) 50 μM cytochrome c reduced with dithionite; (c), 100 μM pHPP; (d), summation spectrum obtained as (b)+(c); (e), 50 μM ferric cytochrome c + 100 μM pHPP recorded 20 min after mixing; (f) subtraction spectrum obtained as (d)-(e). The spectral analysis is representative of 3 independent experiments yielding similar results. (**C**) Oxymetric traces. Isolated beef heart mitochondria (BHM) where added to the medium of (A) at the final concentration of 0.5 mg prot/ml and cytochrome c added to reach the indicated final concentrations; where indicated 5 mM NaN_3_ were added. The shown oxymetric traces are representative of 3 independent experiments; the graph on the left shows the means ± SEM (n = 3) of the OCRs attained at the indicated concentrations of cytochrome c and corrected for the NaN3-insensitive activity; the P value was < 0.01 for each data point in the presence of pHPP *vs* the corresponding CTRL.

Finally, we sought to verify the redox properties of pHPP under our experimental conditions. While testing the direct antioxidant activity of pHPP in the assay which monitors the reducibility of ferric cytochrome c in the presence of a superoxide anion generator system (i.e. xanthine plus xanthine oxidase) [35] we noticed that pHPP alone caused direct reduction of cytochrome *c*. Thus we decided to deepen this point. Following absorbance changes at 550 nm, Fig 7A shows that in the presence of 3 mM pHPP ferric cytochrome *c* was rapidly converted in ferrous cytochrome *c* (with a t_1/2_≈ 8 s). Spectral deconvolution analysis documented that the reduction of cytochrome *c* was accompanied with disappearance of the UV spectral feature of pHPP peacking at 295 nm (Fig 7B) [36]. Moreover, addition of graded concentrations of ferric cytochrome *c* to a suspension of isolated mitochondria supplemented with pHPP resulted in a progressive increase of the NaN_3_-sensitive OCR significantly higher than that attained in the absence of pHPP (Fig 7C and 7D). To notice, even in the absence of exogenously added cytochrome *c* the mitochondria-mediated OCR was higher in the presence of pHPP indicating the capability of the compound to directly transfer electrons to the endogenous mitochondrial cytochrome *c*.

## Discussion

**Fig 8:**
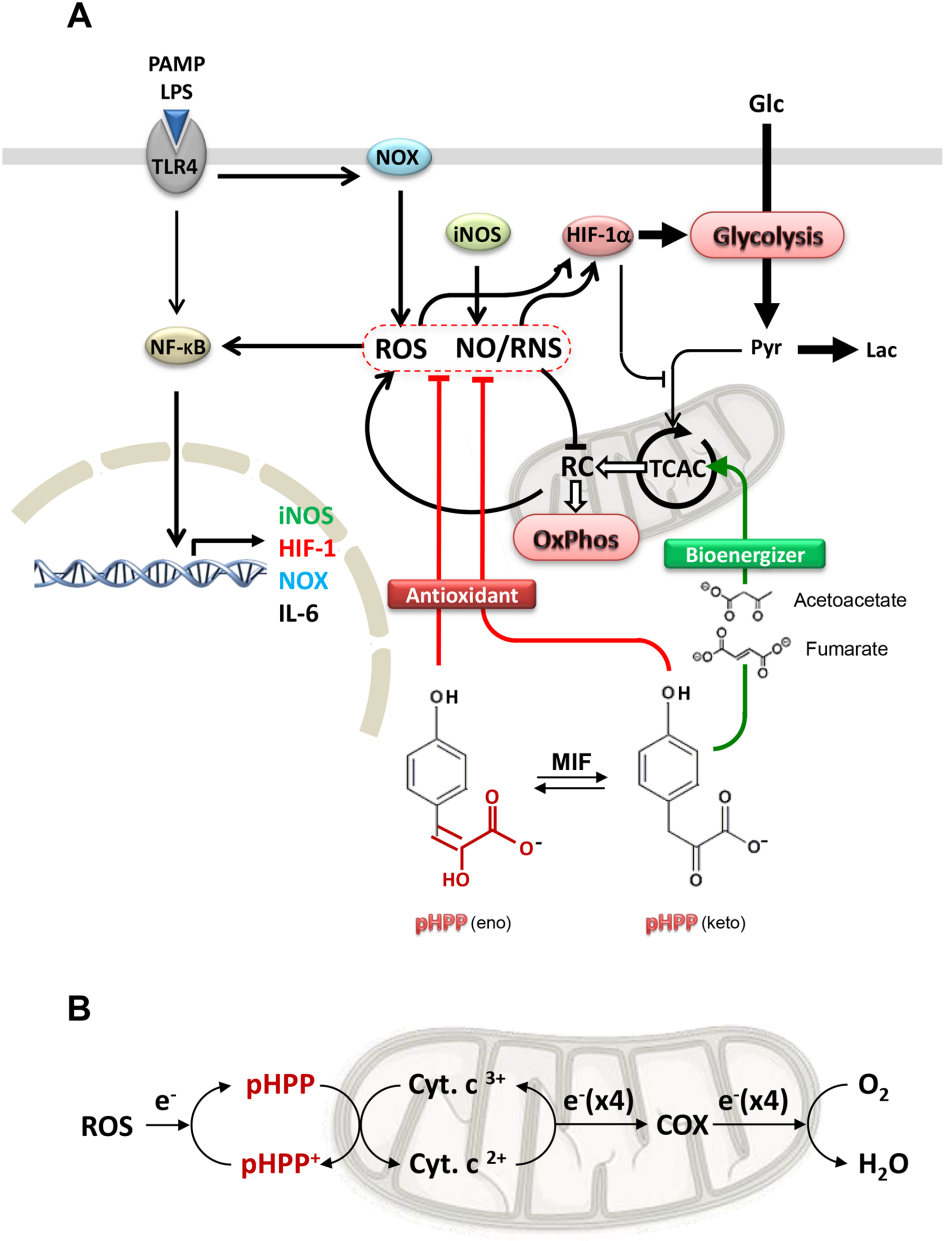
Schematic drawing of the anti-oxidant-mediated anti-inflammatory effect of pHPP. **(A)** The scheme highlights the effect of pHPP as resulting from its combined reactive species scavenger and respiratory substrate-supplier actions. See Discussion for details. PAMPS, pathogen associated molecular patterns; LPS, lipopolysaccharide; TLR4, toll-like receptor 4; NOX, NADPH oxidase; iNOS, inducible nitric oxide synthase; HIF-1α, hypoxia inducible factor 1 alpha; NF-KB, nuclear factor kappa-light-chain-enhancer of activated B cells; ROS, reactive oxygen species; NO, nitric oxide; RNS, reactive nitrogen species; RC, mitochondrial respiratory chain; TCAC, tricarboxylic acid cycle; OxPhos, oxidative phosphorylation; MIF, macrophage migration inhibitory factor. (**B**) Schematic representation of the redox buffering activity of pHPP. COX, cytochrome c oxidase; pHPP^+^, oxidized pHPP.

In a previous study we tested the therapeutic potential of pHPP in a rat model of hemorrhagic shock showing its efficacy in prolonging animals' survival. In the same study we further demonstrated that pHPP displays antioxidant and energetic substrates-supplying properties in a cultured endothelial cell line under stressing conditions [14]. The purpose of that investigation was to screen analogs of EP, which in spite of its cytoprotective action in a number of *in vitro* and *in vivo* animal models of organ damages, failed to show benefit in preclinical trials in human patients [10,13]. In keeping the well-documented anti-inflammatory action of EP we sought to establish whether a similar property was shared with pHPP. To this aim we used the well-established in vitro model of the LPS-challenged macrophage cell line RAW 264.7 [21]. The results obtained allow to propose the working model schematically shown in Fig 8A and detailed onward.

Macrophages play a pivotal role in the innate immunity and respond to local immune- and or pathogen-derived signals to adopt different activation states. Toll like receptor (TLR) agonist such as LPS promotes activation of macrophage from a quiescent to an activated state M1 [37–39]. From the host-defense standpoint, M1 macrophages are inflammatory, secreting mediators such as IL-6, which activates other immune cells, and NO, which is directly cytotoxic, by upregulation of iNOS. Activation of the transcription factor NF-κB is long known to be the major down-stream target of the TLR4-related signaling pathway [40]. The results of the presented study show that, like EP, pHPP was able to prevent the IL-6 release in LPS-treated macrophage but with a much greater efficacy (Fig 1C). It is worth nothing that pHPP did not prevent the M1-polarized morphological changes induced by LPS. This could be related to an LPS-mediated activation of a pathway leading to cytoskeleton remodelling, which is pHPP-insensitive. Alternatively, pHPP could prime polarization of the macrophages from the M0 state towards an alternatively activated anti-inflammatory/immunosuppressive phenotype (M2). Indeed, pHPP-treatment of unstimulated RAW 264.7 cells caused the appearance of elongated spindle-like shaped cells [41]. Analysis of the level of expression of specific M2 markers would help to clarify this point. Even more effective was pHPP in preventing the expression of iNOS and the production of NO as compared with EP (Figs 2 and 3). Moreover, pHPP was able to substantially prevent the over-production of ROS, which is known to accompany the LPS-mediated pro-inflammatory response in macrophage as well as in other immune cells [42]. A direct interaction of the activated macrophage TLR4 with a member of the NADPH oxidase (NOX) family has been shown to be an early event in the LPS-induction of ROS and activation of NF-KB [43]. Consistently, we observed alteration of the redox state and enhanced mitochondria-unrelated OCR in LPS-treated RAW 264.7 (Figs 4 and 5B). However, also dysfunctional mitochondria are recognized as a major source of ROS under condition of pro-inflammatory priming [44,45]. This may well be a consequence of the direct inhibitory effect of NO, competing with O_2_, on the cytochrome c oxidase (CxIV), the terminal electron acceptor of the mitochondrial respiratory chain [46]. The resulting slow-down of the electron transfer rate through the respiratory chain would ensue in accumulation of redox intermediates up-stream of CxIV and enhanced electron leakage producing ROS [47,48]. In addition to compete with O_2_ to the active site of CxIV, NO was shown to inhibit CxIV, as well as CxI, *via* nitrosative covalent modifications [26,49]. This scenario might also include a functional interaction between NOXs and mitochondria via a ROS-induced ROS release (RIRR) mechanism whereby ROS generated by NOX would cause further production of ROS by the mitochondrial respiratory chain [50,51]. The resulting pro-oxidative state is thought to directly activate redox-sensitive transcription factors, like NF-KB [52], priming immune cells to pro-inflammatory commitment.

Consistent with this model we found a significant inhibition of the mitochondrial respiratory activity in intact RAW 264.7 challenged with LPS and of both CxI and CxIV enzymatic activity (Fig 5A and 5D). This latter result, being attained in solubilized cells under normoxic setting, would rule out the competitive inhibition of NO on CxIV suggesting instead a permanent (nitro)oxidative modification of the respiratory complexes. It is worth noting that primary human fibroblasts treated directly with septic sera recapitulated the depressed respiratory phenotype observed with the LPS-challenged murine macrophage cell line (Fig 5C) highlighting the general relevance of this observation. Metabolic flux analysis by SeaHorse technology revealed that the decreased mitochondrial OxPhos activity was accompanied with a compensatory up-regulation of the aerobic glycolysis (Fig 6A–6C) somehow recalling a Warburg-like effect. A major transcription factor controlling the expression of the glucose transporters as well as of the glycolytic enzymes is the transcription factor HIF-1 [53]. Although the alpha subunit of HIF-1 is rapidly degraded following its hydroxylation by O_2_-dependent prolyl hydroxylases (PHDs) [54], hypoxia-independent stabilization of HIF-1α has been amply described [55]. ROS and RNS, as well as intermediates of the Krebs cycle, proved to inhibit the PHDs thereby activating HIF-1 also under normoxic conditions [56–60]. Moreover, activated NF-KB was linked to enhanced transcription of the HIF-1α coding gene *HIF1A* [61–63]. Accordingly, stimulation of HIF-1 hallmarks the inflammatory activation of several immune cells and it is thought to spur rewiring of their metabolic phenotype [64]. Consistent with this notion we confirmed in LPS-stimulated RAW 264.7 a significant stabilization of HIF-1α as compared with the unstimulated resting macrophages (Fig 6D).

Notably, pHPP inhibited all the above described alterations in LPS-stimulated RAW 264.7. Indeed the metabolic shift toward a glycolytic phenotype was completely prevented by pHPP with both the cellular respiration and the respiratory chain complexes I and IV activity displaying no significant changes as compared with the unstimulated cells (Figs 5 and 6). Likewise, the stabilization of HIF-1α was also inhibited.

It must be noticed that all the above-reported effects of pHPP were attained at the low mM concentration range of the compound (i.e. 3 mM). This setting might be considered relatively high in terms of its pharmacological utilization. However, RAW 264.7 cell viability was not significantly affected up to 10 mM of the compound (Fig 3A) and in our previous study [14] 2.5 mM of pHPP resulted in growth stimulation of EA.hy 926 endothelial derived-cells. In the same paper it was shown that administration of pHPP in rats to a calculated final concentration in the circulating blood of about 7 mM resulted in significant delayed death following severe hemorrhagic shock as compared with untreated controls [14]. Consistently, pHPP resulted beneficial in a rat model of multivisceral ischemia and reperfusion where it was administered at a dosage of 0.86 mMol/kg corresponding to about 13 mM of the compound in the circulating blood [65]. To notice for the pHPP-analogue EP a safety level in humans of 150 mg/Kg was established and the drug cyclically injected at a blood concentration higher than 10 mM in high-risk patients undergoing cardiac surgery with cardiopulmonary bypass [13].

Based on the presented results our study clearly shows for pHPP a potent anti-inflammatory activity combined with the ability to modulate the metabolic phenotype in stimulated macrophage. Mechanistically we propose that the effects of pHPP can be mainly attributable to its scavenging activity of reactive species. The pyruvoyl mojety present in pHPP can indeed react with peroxides leading to decarboxylation according to the reaction:

$$\text{R}-{\text{CH}}_{2}-\text{CO}-\text{COOH}+{\text{H}}_{2}{\text{O}}_{2}\to \text{R}-{\text{CH}}_{2}-\text{COOH}+{\text{CO}}_{2}+{\text{H}}_{2}\text{O}$$

where R is the phenol radical. However, the following further possibility can be offered.

Macrophages constitutively express the inflammatory cytokine macrophage migration inhibitory factor (MIF), which plays an important regulatory role in innate immunity through the suppression of anti-inflammatory effects of glucocorticoids [66]. Intriguingly, in addition to its immunomodulatory function, MIF is endowed with an enzymatic activity catalyzing tautomerization of pHPP. The biological function of such a catalytic activity of MIF is cryptic since the Km for pHPP is in the millimolar range (i.e. 2.4 mM [67]) whereas the plasma concentration of pHPP is in the sub-micromolar range (i.e. 0.38 μM [68]). Irrespective of the function, if any, the tautomerization activity of MIF might have fortuitously provided additional antioxidant properties to the administered pHPP under the experimental conditions of this study. Indeed, the enol tautomer of pHPP is expected to extend the delocalization of the π electrons of the aromatic phenol ring conferring to the enol tautomer the ability to scavenge one-electron unpaired species such as O_2_•^-^, OH•, NO•. Accordingly, two compounds, 2-acetamidoacrylate and its methyl ester, which mimics the enol structure of pyruvate (or EP), have been demonstrated to have anti-inflammatory properties both in vitro and in vivo. In particular, methyl 2-acetamidoacrylate is at least 100-fold more efficient than EP to suppress LPS-induced NO production by RAW 264.7 cells [69].

In addition of the afore-mentioned properties this study suggests an additional modality for the antioxidant activity of pHPP (Fig 7A–7C). This may consist in a one-electron transfer to cytochrome *c*, which then delivers it to the cytochrome *c* oxidase reducing O_2_ to water. The oxidized form of pHPP can then be re-reduced by free radical reactive species thereby functioning as a functional redox buffer (Fig 8B). Indeed, the cytochrome c oxidase activity is linked to generation of a protonmotive force that enhances the coupling efficiency between oxygen consumption and ATP synthesis.

Accordingly with our hypothesis a comparative study of the effects of EP and α-keto carboxylic derivatives including pHPP in a rat model of multivisceral ischemia and reperfusion showed a superior antioxidant activity of pHPP in reducing the liver content of malondialdehyde an oxidative stress marker [65].

In addition to the antioxidant properties of pHPP it has to be considered its bioenergizing function. pHPP is an intermediate of the catabolic pathway of phenylalanine/tyrosine leading to acetoacetate and fumarate as end products in the cytosol. In our previous study we showed that pHPP markedly induced oxygen consumption in isolated mitochondria only in the presence of the cytoplasmic fraction [14]. Both acetoacetate and fumarate, by the monocarboxylate and dicarboxylate carriers respectively [70,71], can enter into the mitochondria and fuel the Krebs' cycle. This would bypass in the LPS-stimulated macrophage the impeded oxidative entry of pyruvate in the terminal catabolism proved to be caused by the HIF-1-mediated expression of the pyruvate dehydrogenase kinase, an inhibitor of the pyruvate dehydrogenase activity [72].

In conclusion all together the presented results point to the *in vitro* observed superior anti-inflammatory action of pHPP as a combination of effects. These comprise on one hand a broad antioxidant activity likely enhanced by the MIF-mediated keto-enol tautomerization of the pyruvoyl mojety of the molecule, and on the other hand the capacity to deliver catabolic end-metabolites that fueling the mitochondrial Krebs' cycle and the OxPhos machinery counteracts the glycolytic metabolic rewiring of activated immune cells.
